# Building digital resilience: leading healthcare transformation through an online community

**DOI:** 10.3389/fdgth.2025.1656804

**Published:** 2025-09-05

**Authors:** Nirit Putievsky Pilosof, Yaara Welcman, Michael Barrett, Eivor Oborn, Stephen Barrett

**Affiliations:** ^1^ Coller School of Management, Tel Aviv University, Tel Aviv, Israel; ^2^ Cambridge Digital Innovation—CJBS & Hughes Hall, University of Cambridge, Cambridge, United Kingdom; ^3^ Cambridge Judge Business School (CJBS), University of Cambridge, Cambridge, United Kingdom; ^4^ Warwick Business School, The University of Warwick, Coventry, United Kingdom; ^5^ Auckland City Hospital, Auckland, New Zealand

**Keywords:** healthcare transformation, digital resilience, online community, leadership, collaboration, digital technologies, qualitative study

## Abstract

**Introduction:**

Healthcare systems globally face systemic vulnerabilities, such as crisis response, insufficient capacity, lack of integration, and rising care costs while simultaneously being pressured to accelerate the shift toward digital health solutions. In response, new organizational forms and digitally enabled collaborations have emerged to support care continuity and innovation. This study examines how digital resilience can be built at a system level through a national online community of healthcare professionals. Drawing on a longitudinal qualitative case study of Israel's Digital Health Community, an initiative launched by the Ministry of Health in 2020 in response to COVID-19 crisis, we explore how a digitally mediated, cross-sectoral online community with more than 1,200 medical professionals from various disciplines and organizations enabled national healthcare transformation through digital resilience.

**Methods:**

Using interviews, observations, and digital document analysis conducted over four years, we trace how the online community enabled systemic resilience through three interconnected dynamics: the redefinition of roles and responsibilities across disciplines, enhanced collaboration across organizations and governance levels, and the development of a culture of innovation.

**Results:**

By challenging existing norms, the online community facilitated an entrepreneurship approach, fostering leadership in healthcare transformation and overcoming professional resistance to change. These interactions helped generate integrated models of care, informed national digital health regulation, and enabled rapid experimentation in service design and delivery. We argue that digital resilience plays an important role in enabling these healthcare transformations.

**Discussion:**

We present a conceptual model that illustrates how digital resilience is produced not as a fixed organizational trait, but as an emergent, multi-level outcome of structured community engagement. It highlights the need for new governance models that merge top-down and bottom-up involvement and leadership, moving from hierarchical to network structures to diffuse innovation and transformation among diverse stakeholders across the healthcare ecosystem.

**Conclusions:**

Our findings contribute to the growing literature on digital health transformation by highlighting the role of participatory, networked approaches to resilience-building. The study offers actionable insights for policymakers and health system leaders seeking to institutionalize adaptive capacity through digitally enabled collaboration.

## 1 Introduction

Global healthcare systems are facing growing rapid challenges, ranging from data breaches and workforce shortages to pandemics, severe overcrowding and technological disruptions (1–4). These pressures have revealed systemic vulnerabilities and emphasized the urgent need for more adaptive, responsive, and future-ready infrastructures (5, 6). In this context, digital resilience has gained growing attention as a pathway to sustainable healthcare transformation (7). Digital resilience refers to the ability to proactively respond to disruptions by leveraging digital tools, infrastructures, and collaborative networks to sustain and improve healthcare delivery (8, 9). While resilience more generally refers to the ability and capability to absorb and adapt to shocks (10), digital resilience focuses on how digital technologies can achieve these outcomes as well as the holistic process of developing more of such system-wide capacities.

Although healthcare systems have invested in digital technologies, the integration of these tools into clinical and organizational routines remains inconsistent. Persistent barriers such as professional silos, regulatory misalignment, and organizational inertia often hinder transformation (11–14). Moreover, much of the literature on digital resilience focuses on the adaptation of individuals or single organizations, rather than examining how resilience emerges at the system level through shared infrastructures and participatory governance.

This study addresses that gap by investigating how digital resilience is co-produced through the everyday practices of a national online community of healthcare professionals in Israel. We explore how a digitally mediated professional community supports digital resilience across three interrelated dimensions: the development of digital abilities (skills and practices), the emergence of system-wide capabilities (shared norms, coordination, and innovation mechanisms), and the expansion of overall capacity (the scalable potential to absorb shocks and reconfigure care delivery) (Table 1).

**Table 1:** Ability, capability, and capacity perspectives on digital resilience in the context of healthcare [adapted from (9)].

Term	Definition	In explaining resilience	Healthcare example
Ability	A specific skill or trait possessed by a person or group.	The foundational enablers of resilience, without necessarily expanding on how the abilities are orchestrated to enable resilience.	The ability of healthcare professionals to acquire digital proficiency enables them to use digital platforms and tools independently or collaboratively.
Capability	Combined competencies formed by pooling resources and skills.	The combined competencies of a system, resulting from pooling resources and abilities, that are needed to enable resilience.	The capability of healthcare managers to coordinate professional expertise, digital tools, and collaborative networks to maintain operations during crisis.
Capacity	The maximum potential of a system to absorb, adapt, and transform.	The limits of a system's resilience. Capacity extends beyond the mere presence of specific capabilities. The conditions of a system when shocks occur, the recovery needs, and how capabilities are deployed and leveraged.	The full extent of a healthcare system to scale, innovate, and reorganize, by implementing system capabilities, using digital infrastructure and networks in response to the specific nature of the shock encountered.

Our research question is: How does an online community contribute to the development of digital resilience in a national healthcare system? Drawing on longitudinal qualitative data collected between 2020 and 2024, including interviews, digital communications, and observations, we offer a conceptual model that shows how online, cross-sectoral collaboration can foster system-wide transformation. Our findings contribute to emerging literature on digital health by demonstrating how participatory, digitally enabled communities can build healthcare systems that are not only reactive but adaptive, innovative, and a process through which they become future-ready.

### 1.1 Digital resilience in healthcare

Resilience has traditionally been defined as the ability of systems, organizations, or individuals to absorb shocks, recover, and maintain core functionality under stress (15–17). In healthcare, this concept has been expanded to capture not only the capacity to recover but also to adapt and transform in response to emerging challenges, such as those posed by pandemics, demographic pressures, or technological disruptions (18). The COVID-19 crisis emphasized the urgency of developing more flexible and responsive healthcare infrastructures that can withstand systemic stress and reconfigure in real time.

Digital resilience has emerged as a related but distinct concept, defined as the ability to proactively respond to external shocks by leveraging digital tools, infrastructures, and collaborations (8, 9). This includes the strategic deployment of digital platforms, the integration of automation and analytics, and the cultivation of new forms of organisational coordination. Mahmood et al. (19) propose a dynamic framework for digital resilience that includes preparation, crisis absorption, adaptive transformation, and ongoing evaluation, emphasizing that resilience is an ongoing process, not a one-time achievement or outcome. As such we link resilience with healthcare transformation as a response to system shocks.

Most of the empirical work in this area, however, remains focused on individuals or specific organizations: for instance, how physicians adapted to telemedicine during the pandemic (20), or how digital platforms helped companies maintain operations (21). Complementing this perspective, Kaligis et al. (22) demonstrate how structured digital mental health programs supported transitional-aged medical students, offering evidence that online platforms can foster resilience both at the individual and institutional levels. Similarly, Wekerle et al. (23) highlight youth-led digital resilience tools, such as the JoyPop™ app, which illustrate the capacity of digitally mediated environments to scaffold self-regulation, adaptation, and community engagement across complex systems.

These studies point to a growing recognition that digital resilience emerges not solely from personal coping strategies or isolated organizational resources, but from networked and participatory infrastructures. While useful, they underscore the need for further research at the system level, where resilience is co-produced across institutional boundaries through collaboration, learning, and digitally mediated interactions.

### 1.2 Online communities of practice

Digital communities are evolving environments in which identity significantly influences member interaction and engagement (24). These online spaces develop shared interests through participatory processes. The community analyzed in this study exemplifies a digitally mediated community of practice (25), a structure that has gained recognition as essential for organizational learning and knowledge exchange (26). These communities evolve based on internal dynamics such as leadership and participation, as well as external conditions including technological changes, institutional regulations, and sector-wide competition. By enabling dialogue, storytelling, and peer recognition, online communities offer healthcare professionals a forum for constructing meaning around their work. The open and flexible nature of these platforms allows for diverse levels of engagement and reflective practice (27).

There is growing recognition that communities of practice, especially those organized around digital platforms, may play a vital role in enabling system-wide adaptability. Online professional communities allow geographically dispersed individuals and institutions to collaborate, share knowledge, and collectively develop solutions, often more rapidly and transparently than through traditional hierarchical channels (28, 29). Such communities can act as neutral spaces for innovation, particularly in healthcare systems where providers may compete in other contexts (30, 31).

Moreover, digitally mediated communities facilitate boundary-spanning interactions across organizations, professions, and regions, supporting knowledge recombination and the co-creation of innovative practices (32, 33). Through these interactions, abilities and skills linked to using new digital technologies in practice could be enabled across online communities. This flexibility is particularly important in dynamic healthcare environments where rapid changes in technology, policy, or clinical needs require coordinated yet agile responses.

## 2 Methods

This qualitative study investigates how digital resilience is developed within a national online community of healthcare professionals, focusing on the dynamic interplay between professional, organizational, and system transformation. We adopt an interpretive research paradigm, viewing resilience not as a static trait but as a relational and evolving capacity enacted through interactions among individuals, technologies, and institutions (9, 18).

This study, designed to investigate the development of the Digital Health Community and its impact on the healthcare ecosystem, was conducted from November 2020 to August 2024, with full access to the community activities and documentation. Our data collection process involved detailed observations of numerous online community meetings, webinars, WhatsApp group communications, and social media engagements. We also conducted twenty five semi-structured interviews with community members and representatives from the MoH, of 30–60 min, which were transcribed and translated into English (Table 2). After completing the data collection, we employed a thematic qualitative data analysis methodology. This methodology identified emerging themes based on principles of naturalistic inquiry and a grounded approach to conceptual development.

**Table 2:** Data collection for the study of the Israel digital health community.

Data type	Description	Quantity between 2020 and 2024
Semi-structured Interviews	Interviews with diverse professionals from various roles and healthcare organizations (physicians, therapists, innovation managers, MoH representatives). Names anonymised for confidentiality.	25 interviews.
Participant Observation	Online Zoom meetings of the working groups, accelerator sessions, and the community conference.	28 meetings, last on average 90 min. The community conference was full day event.
Meetings with the MoH team	Documentation of group meetings with the MoH team to present emerging findings, validate themes, and receive feedback.	5 meetings.
Webinars (recordings and transcripts)	Webinars featuring selected presenters and community members, including the ’successful stories' presentations and the Research Club meetings.	18 webinars.
WhatsApp Messages	Netnographic observations of ongoing Interactions of members in four WhatsApp community groups	Digital traces of message thread over 4 year period. One member of team participated in ongoing conversations.
LinkedIn Posts	Ministry and community members' posts, public announcements and endorsements.	Digital traces of posts with comments and reposts over the final 18 months of study.
Documents, publications and announcements	Calls for innovation proposals, invitations to community events, summaries of discussions, and published guidelines.	All formal community communication has been archived.

**Figure 1:**
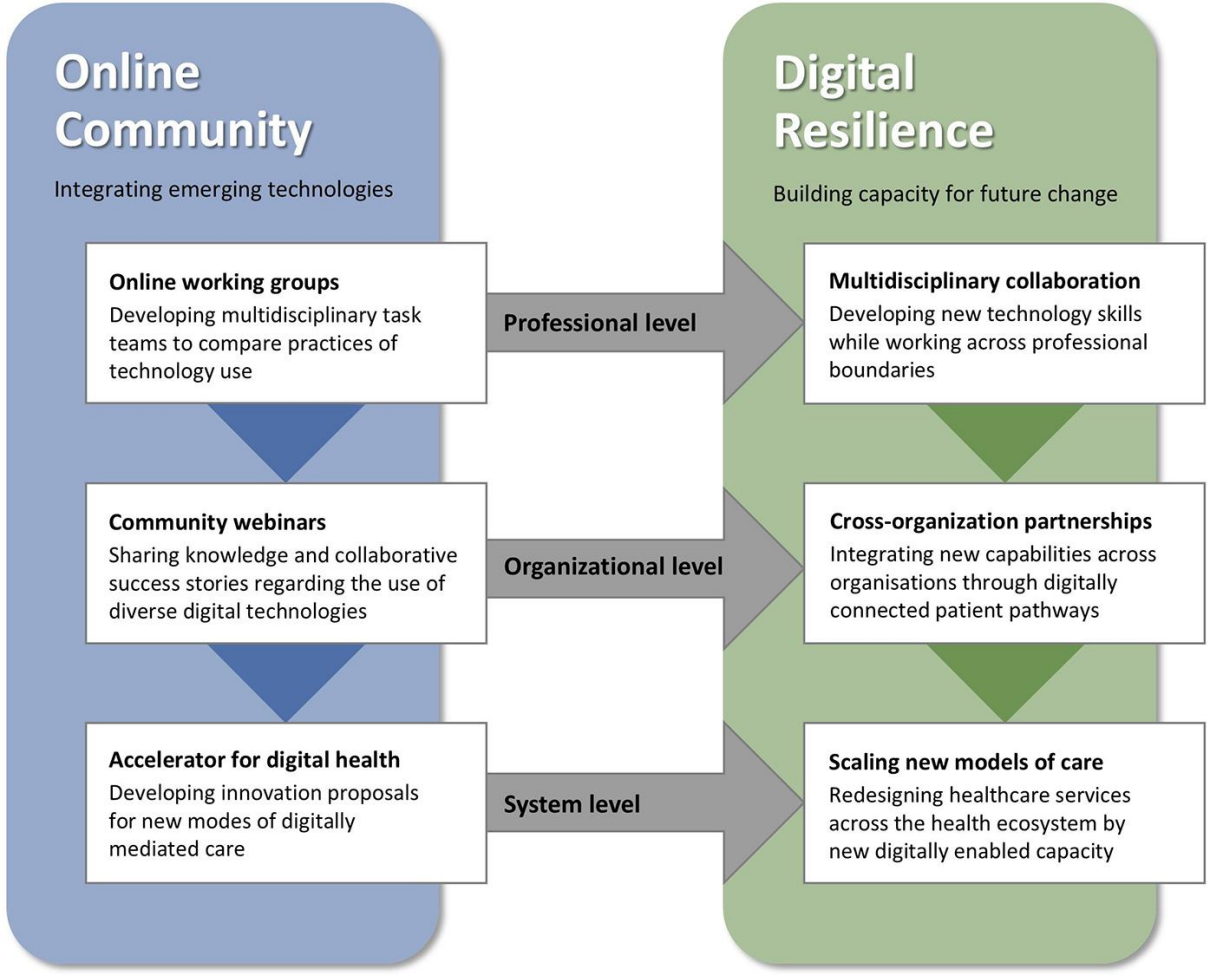
The online community enables digital resilience by changing practices on professional, organizational, and system levels.

**Figure 2:**
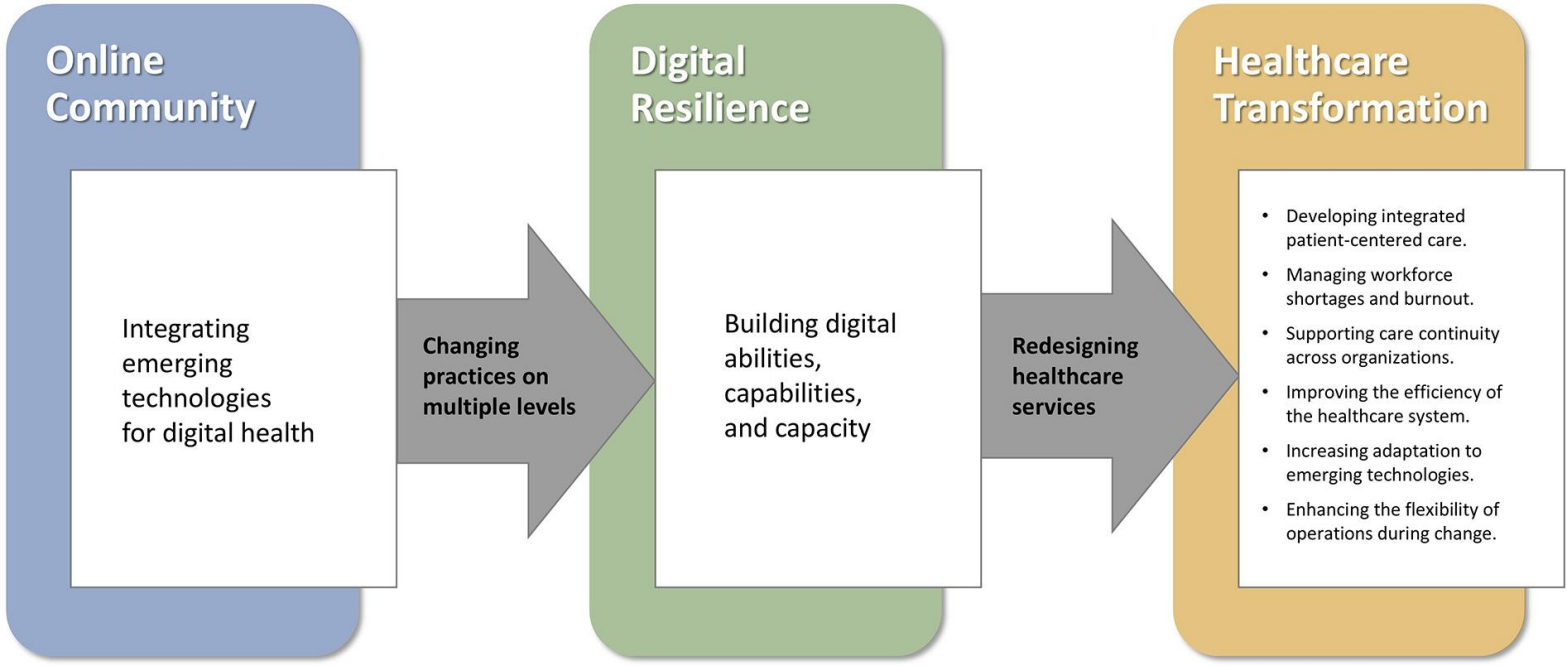
Leading healthcare transformation by building digital resilience through an online community of healthcare professionals.

The study explored how the community's digital interactions did not merely reflect resilience but actively produced it through new configurations of relationships, norms, and digital affordances. Using an analytic approach, grounded in an interpretive epistemology and informed by resilience theory and discourse (34, 35), we developed the analysis in three phases. We began with iterative close reading of the community documents, publications and announcements, interview and webinar transcripts, field notes, and digital artifacts such as discussion threads. In the second phase, we iteratively identified recurring themes linked to three resilience dimensions emerging from the literature: ability, capability, and capacity, and examined how these developed through professional, organizational, and system levels, as outlined in our conceptual framework (Figures 1, 2). In the last phase, we presented the analysis and conceptual framework to the MoH team to discuss the model and learn from their reflections and insights.

## 3 The case of the Israel digital health community

In response to the outbreak of the COVID pandemic in 2020, the Digital Health Division of the Ministry of Health (MoH) launched a digitally mediated “community of practice” designed to promote cross-organizational learning, telemedicine adoption, and health service innovation across the public healthcare system. Although framed as participatory and bottom-up, the initiative was significantly shaped by institutional interests and state reform goals, particularly those emphasizing efficiency, digital fluency, and shifting more care into the community and home using new digital technologies. The MoH, responsible for policy making and regulation of all healthcare organizations through mechanisms of supervision and financing, created and managed the online community of healthcare professionals, with over 1,200 members, including primarily doctors, therapists, nurses, and administrators, with an objective to connect to the “field” and promote bottom-up leadership across the healthcare system. The community, originally focused on remote care expanded its scope to cover broader aspects of digital transformation, resulting in a name change from the “Israel Telemedicine Community,” to the “Digital Health Community” in the post pandemic period. The community provided a platform for interdisciplinary working groups, knowledge-sharing webinars, and an innovation accelerator designed to support the development and scaling of new digital health solutions.

Engagement in the Digital Health Community has evolved significantly over time. During the pandemic's acute phase (2020–2022), participation was intensive and oriented toward urgent problem-solving, with frequent Zoom meetings and continuous WhatsApp exchanges and accelerator projects aimed at rapid deployment of telemedicine and remote care solutions. In the post-pandemic period (2023–2024), activity shifted toward more structured, less frequent interactions focused on broader digital health initiatives for long-term digital transformation, scaling successful pilots, and aligning innovations with national strategies. The community has continued to evolve in response to new crises and the AI revolution.

### 3.1 Online working groups

#### Developing professional groups and multidisciplinary task teams

The community's working groups initially formed around specific clinical domains, including family medicine, mental health, oncology, geriatrics, paediatrics, diet, and rehabilitation. The professional groups were composed primarily of professionals from the same discipline to share knowledge and develop guidelines. Over time, the professional discipline groups evolved into multidisciplinary task teams, recognizing the potential to work across professional boundaries. Members from different professions and organizations collaborated in multidisciplinary task teams to address common challenges, such as implementing telemedicine, managing digital triage, or improving remote patient monitoring.

Meetings were held via Zoom and supported by ongoing WhatsApp conversations, allowing for continuous coordination, informal learning, and trust-building. Participation was voluntary and peer-led yet tightly connected to national health priorities. This structure enabled both grassroots innovation and alignment with system-wide transformation goals. The professional working groups and multidisciplinary task teams functioned as “laboratories” for care innovation, enabling members to co-develop new models of service delivery that responded to emerging needs, particularly those related to workforce shortages, continuity of care, and the integration of digital tools into everyday clinical practice.

Over time, some of the online working groups have decreased or terminated their engagement, mostly when crises seem resolved (such as the end of the pandemic). Given that most of the scheduled meetings were during non-work, evening hours, commitment to the community also produced fatigue and wear on participants. While most group leaders were appointed by the MOH, often according to stature in the relevant medical field, which also could serve the Ministries interest and ability to retain control, some leaders were more effective at nurturing engagement. In several groups, “self-appointed” leaders had the ability to steer group activity. In most cases, the lack of an overall mission and strategic rationale or purpose significantly influenced the ongoing commitment of some groups, with participation declining over time.

### 3.2 Community webinars

#### Sharing organizational knowledge and collaborative success stories

A central mechanism for knowledge exchange in the community was a series of regular webinars. These sessions were designed to share success stories and lessons learned from collaborative projects, which emerged from the community's working groups and task teams. Given the wide range of participants, these were scheduled in the evening and night to simplify maximum diary coordination. Webinars provided a low-barrier, accessible space where professionals from different organizations could present pilot projects, share implementation challenges, and receive feedback from peers and Ministry representatives. Themes included remote care for specific populations (e.g., geriatrics, pediatrics), digital health app evaluations, mental health technologies, organizational strategies for change, and research studies.

The webinars shifted the focus away from organizational affiliation, professional ranking, and academic prestige, emphasizing shared goals, challenges, and solutions. The digital format allowed for broad participation across sectors and regions, mitigating hierarchies and competition between disciplines and organizations, to support the community's role as a national platform for shared learning and responsive system improvement. Engagement in the webinars fluctuated over time, especially after the pandemic crisis settled, mainly depending on the relevance of the topic to community members and their healthcare organizations.

### 3.3 Accelerator for digital health

#### Developing innovative proposals for new models of care

To further support transformative ideas generated within the community, the MoH established an Innovation Accelerator Program. This initiative invited multidisciplinary teams, including participants from different medical organizations and professional backgrounds, to submit proposals for new models of care integrating emerging technologies, fostering digital service innovation. An evaluation committee from the MoH prioritized proposals based on the significance and relevance of the challenge they selected to address. Accordingly, the chosen proposals often addressed system-level challenges such as digital continuity of care, new triage models, or integrated data platforms.

Selected teams from the community were given structured support, including mentorship, design-thinking workshops, training sessions, and access to strategic decision-makers during approximately ten full-day meetings over four months. At the end of the program, the teams showcased their projects and competed for financial support from the MoH to develop and implement their innovative new models. Most of the projects were found eligible for two-year implementation support, but since they are still a work in progress, it is difficult to assess their success rates.

Still, the accelerator became a mechanism for fast-tracking promising initiatives by turning grassroots ideas into implementable projects. It reinforced the evolving identity of online community members as not only clinical experts but also digital innovators. It also institutionalized a space within the community for experimentation and knowledge co-production, contributing to the health system's broader ability to anticipate and adapt to future disruptions.

## 4 Results

This section presents the key mechanisms through which the Israel Digital Health Community contributed to the development of digital resilience on multiple levels: professional level, organizational level, and system level. Figure 1 illustrates how the activities of the online community, incorporating emerging technologies, enhanced digital resilience by transforming practices across the three levels. The online working groups encouraged multidisciplinary collaboration at the professional level. The community webinars promoted cross-organization partnerships at the organizational level, and the accelerator for digital health promoted the scaling of new models of care at the system level. Drawing from interviews, observations, and analysis of online communication, we highlight the interrelated dynamics behind the changes on the three levels, leading to digital resilience through multidisciplinary collaboration, cross-organizational partnerships, and scaling new models of care.

### 4.1 Multidisciplinary collaboration

#### Professional-level change: working across professional discipline boundaries

Integrating digital technologies within the online community has created new forms of interaction across professional boundaries, fostering multidisciplinary collaboration as a key driver of healthcare transformation. Platforms like Zoom and WhatsApp enabled regular, agile online engagement between professionals from diverse specialties, facilitating real-time dialogue and collaborative problem-solving. These digital spaces allowed clinical practitioners from diverse healthcare disciplines, managers, and innovation leaders to co-develop care strategies, bypassing hierarchical structures and professional silos, traditionally fragmenting healthcare [e.g. (36),].

In the online forum, reminders of disciplinary divisions—such as name tags or dress codes—were harder to identify, emphasizing the content of contributions. This fosters an ability to value knowledge beyond one's disciplinary base and extend one's thinking into other domains to make new connections (Online community group leader).

The community supported collaboration among physicians, nurses, and other health professionals, who are trained and often supervised by different management units, in addressing shared challenges in care delivery.

“Originally, the oncology working group focused on [practices of] physicians. When I participated in other groups, I saw how they involved other disciplines and health professions, not only physicians, who utilized communication with the patients and developed new tools. I realized that is very important, and as a result, we tried to bring in oncology nurses into our group discussions.” (working group leader in the community)

As programs evolved to bring together professionals from medicine, nursing, rehabilitation, and digital health, they supported the co-creation of integrated care models that are better aligned with complex patient needs and emerging population health challenges, such as chronic disease management or elderly care.

“We realized (in our geriatric group) that we needed a multidisciplinary team, so the (task group) team was made up of a speech therapist, computer specialist, medical equipment, and an engineer. We even found an ear, nose, and throat doctor from a different hospital to join us”. (Online community group leader)

### 4.2 Cross-organization partnerships

#### Organizational-level change: navigating between competing organizations and hierarchies

Israel's healthcare system is structured around four main Health Maintenance Organizations (HMOs) that provide coverage and services to all citizens under the national health insurance law. The HMOs operate hospitals, clinics, and other facilities across the country. Some hospitals are governmental, some public, and a few private. This multi-stakeholder system creates a complex, competitive environment between the major providers. Moreover, regulations prevent direct problem-solving discussions between the MOHs, which could conflict with competition rules.

“The modus operandi of HMO administrations is based on competition between the HMOs. Furthermore, according to regulations, the Ministry of Health cannot call us in to sit together and discuss our problems with them because that is antithetical to the rules of competition”. (Online community group leader)

While competition among healthcare organizations often drives improvement in healthcare services quality and accessibility, it is often a barrier to building resilience on a national scale. The community allowed for a reduction in competition between organizations by prioritizing collaboration and learning over competitive tensions. The virtual format of the community Zoom meetings simplified logistical complexities of in-person meetings, such as choosing locations, hosting, and representation. It allowed members from different organizations to collaborate more easily as the virtual environment and the online meeting format also enable members to engage without displaying their backgrounds or organizational logos, focusing instead on shared professional goals and collaboration.

“At that point, the sharing was more important than the organizational competition. (…) Try to bring the senior management of the Department of Computers and Innovation of the Community based Healthcare delivery organizations into one room—it is an almost impossible task (…): Where are we meeting? Who is hosting? Who sends which representative? Be careful not to mention the new project we are doing in this area. The community that was established, and the fact that it is virtual, by ZOOM, glosses over a lot of complications in such events.” (family doctor and a community member)

The community working groups, webinars, and the accelerator program enabled collaboration between competing organizations, exemplified by doctors from different institutions jointly developing guidelines for geriatric and pediatric remote care. Additionally, nurses from a hospital in the north district collaborating with a hospital in the center highlight the potential for cross-organizational partnerships.

In the community, caregivers from different medical organizations also came together with regulatory representatives from the MoH. Many emphasize how, while interactions with regulators typically involve insincere posturing, the community stands out for its authenticity in flattening hierarchies:

“(…) If representatives of other organizations are present (…) you try not to present anything that will make a wrong impression (…) Here, in the group (…) There was a very true sense that the regulators' representatives really wanted to understand and really want to implement change (…) and that resulted in a very real desire to cooperate, because maybe this cooperation, together Ministry of Health's resources and tools, will attain something that could not be achieved by the organization alone”. (Online community group leader)

### 4.3 Scaling new models of care

#### System-level change: redesigning healthcare services across the healthcare ecosystem

The ability to scale new models of care is essential for building system-level digital resilience. In an increasingly complex and dynamic healthcare environment, the capacity to manage change by redesigning and scaling new solutions proactively is critical for long-term sustainability. The online community provided a unique structure to support this capacity, offering a platform where healthcare professionals could evolve from traditional clinical roles into innovative leaders within their organizations and the broader healthcare system.

The online community enables experimentation with new tools, allowing members to integrate technological advancements into their services and redesign their practice. This shift is captured by a nutritionist who emphasized, “We need to see how we can leverage these tools,” highlighting the importance of transformation by using new digital innovations.

“We are planning to redesign healthcare services. It is not just to bring some decision-support system to help the GP with their work, which can benefit the patient and the physicians and be more efficient and economical. That is not enough. Currently, GPs are treating about a thousand patients, but in ten years, they will have to treat six or ten thousand patients. How will they be able to do it? For that, we need to rethink and redesign the process completely.” (Representative of the Digital Health Division at the MOH).

Accordingly, the community's support structure allows healthcare professionals to develop and scale new models of care. By providing a space for sharing knowledge and experiences, the community enables its members to stay ahead, continuously improving their practices and adapting to new challenges as they learn from each other:

“…there’s a lot of learning and sharing of information inside the community that surely helps when you come to develop a new service. And you have someone to call, and probably someone did it before you.” (Ministry of Health representative)

The community platform of working groups, webinars, and the accelerator program also enables scaling local initiatives and successful pilots of new models of care across the healthcare system.

“I presented my project [at a MoH program], which was a mobile clinic, and the head of innovation for our HMO was in attendance. When he heard about it, he said, “Great idea.’ We joined [to the community's accelerator] me, the otolaryngologist, the GP from that area who knows the patients and the problems, and our head of administration… and it was like learning how to build a startup… Today, we have three mobile clinics operating in very rural areas in the South. We offer otolaryngology and dermatology services, and we're continuing to expand.” (Otolaryngologist and community member)

The involvement of the MoH within the community supports the ability to scale initiatives developed by the community members.

“We can do great things between us in the working groups of the community, but if we want to scale our projects, like make our [organizational] guidelines national so all the organizations in the healthcare system will use it, we also need a top-down approach. … having round tables with all the stakeholders. We need their help to stimulate and promote the ideas that comes out of the online community groups”. (Online community group leader)

This perspective emphasizes grassroots innovation as the foundation for long-term resilience. By enabling caregivers to lead in proposing and piloting digital solutions, the community created a collaborative and adaptive space that encouraged learning from successes and failures. This ground-up experimentation was critical during the pandemic but has also proven essential in preparing the system for future disruptions. A representative from the Ministry of Health described the effectiveness of this strategy during times of crisis:

“In the following crisis, the community infrastructure was found to be efficient in adaptations. When we needed to adjust the system, the community provided flexibility to develop new models and ways to provide healthcare services at scale.” (Ministry of Health representative)

## 5 Discussion

The Digital Health Community has built digital resilience in the health system, enabling transformation of healthcare services. As summarized in Figure 2, transformation is enabled by learning to integrate digital technologies at multiple levels—with clinicians developing skills in their professional practice, organizational divides becoming minimized through ongoing knowledge sharing and integration across levels between users at the practice level and policy makers at the system level. Enabling digital resilience through improved digital abilities of the workforce, heightened digital capabilities across health services, and increased digital capacity for system-level resilience was foundational for transformation of the healthcare system. The transformation further supported and fueled the augmented digital resilience, which in a virtuous circle also enabled future-proofing the healthcare system to withstand future shocks and challenges. As such, our findings suggest that developing digital resilience at multiple levels helps healthcare entities not only to bounce back in response to crises and shocks, but rather to bounce “forward” for healthcare transformation (37).

Figure 2 illustrates how the online community, integrating emerging technologies for digital health, enabled digital resilience by changing practices on multiple levels (professional, organizational, and system levels). Building digital resilience of abilities, capabilities, and capacity supports redesigning healthcare services to lead healthcare transformation. The figure presents a few examples of healthcare transformation in developing integrated patient-centered care, managing workforce shortages and burnout, and more.

The online community more specifically provided mechanisms to span multiple levels of engagement: from individual role changes and multidisciplinary coordination to organizational alliances and national-level innovation scaling strategies. By starting with multidisciplinary collaboration, the community facilitates knowledge integration across traditional medical silos (36, 38) hereby improving adaptability to new ways of working. Over time, the online community fostered the creation of integrated care models that address complex patient needs by combining clinical, technical, and managerial expertise in new ways. Thus, while change at the level of professional practice is often stymied through resistance (39–41), the online community enabled conversations and learning between clinicians in an unthreatening manner so clinicians could learn new perspectives on technologies used in practice from each other. In the long run, these capacity building measures help to manage workforce shortages and prevent burnout, which are major global challenges (20, 42, 43).

Concerning digital abilities and capabilities, the discussions and implementations of new digital technology in care processes developed clinicians' abilities to work with technology and enabled improved digital resilience. Experience in using digital tools brings confidence and helps reinforce safety by improving care quality, streamlining workflows, and reducing errors through enhanced information access and decision support (44). At the service delivery level, cross-organizational partnerships, involving new ways of working across boundaries to deliver care pathways in more cooperative ways, such as psychiatrists joining family doctors to support rapid access to mental health services at a distance, were enabled by technologies which spanned coordination between previously siloed institutions. As services develop new capabilities using technologies, old patient pathways can be questioned, rearranged and ultimately improved to cope with an evolving crisis. While the development and integration of new services is often a slow process (41, 45) fraught with inertia (39, 46), new digital capabilities can aggregate resources into innovative configurations (3, 38, 44, 47–50).

Cross-organization partnerships also improve care continuity for patients between healthcare providers and institutions and enhance the overall efficiency of the healthcare system. The ability to scale new models of care through the community infrastructure, supporting the rapid expansion of innovative services, is evidence of digital resilience at the system level, with capacity and readiness for future disruptions. By enabling digital capacity of the system, the online community served as an impetus for redesigning healthcare services, as emerging technologies were adopted and adapted to enhance the flexibility of operations on a national scale in response to the crisis. In many national contexts, policy makers are removed from the realities of emerging grassroots clinical concerns (51) or working to a different timeline of priorities (52). The disconnect hampers a timely response to crisis and system shocks, with important stakeholders left unaligned. In our study, we found that the MoH direct involvement in seeding and supporting innovative technology development at the system level within the incubator context allowed them to scale emerging new practices into national guidelines or specific resource allocation.

The mechanism for community transformation at multiple levels was enabled through the use of remote communication and social media technology. WhatsApp, Zoom, and LinkedIn, provided an inexpensive neutral space for connection and collaboration among community members from competing organizations, including the ministry's regulatory officials. This was especially relevant during the challenging era surrounding the rapid establishment of the community amid the COVID-19 pandemic. Additionally, technology allowed for the convenience of meeting from diverse locations, which is important to support time efficiency, given the busy schedules of all stakeholders involved. Therefore, technology has been an essential factor in the ensuing transformations within the community, adapting to and navigating during and after the pandemic crisis. These digital technologies worked well with the online community as a digital space for redesigning healthcare services, where members collectively imagined, discussed, and shaped future scenarios on issues relevant to the community. Through storytelling, debate, brainstorming, and co-writing, participants critically assess current realities and creatively construct alternative futures they agree on for future development.

Although the mechanisms of the community are social at their core, our findings demonstrate that the digital affordances of the online community shaped its operation and impact. Digital tools such as Zoom enabled synchronous engagement among geographically dispersed actors without the logistics and costs of travel. WhatsApp allowed for persistent, asynchronous exchanges that preserved the continuity of dialogue, captured decision trails, and enabled rapid peer support outside scheduled meetings. LinkedIn extended the reach of the community's outputs, providing public recognition that reinforced professional legitimacy and encouraged cross-sector uptake. These affordances did not simply replicate existing face-to-face dynamics online; they reconfigured them, accelerating feedback loops, flattening hierarchies by obscuring visible status markers, and allowing parallel work streams to progress in multiple spaces simultaneously. In this way, the “digital” in digital resilience refers not only to the integration of emerging technologies for digital health but to the community digital platforms that expanded the speed and scale of resilience-building processes.

The involvement of the MoH in engaging with and supporting ideas from community members and groups was significant for the national adoption of projects and guidelines, thereby extending the reach beyond local efforts on a larger scale. It also provided community members with a sense of legitimacy, as they would have an actual influence on the practical arrangement of healthcare services. While bottom-up grassroots involvement of community members in working groups was fundamental for immediate impact and served as a cornerstone for more extensive, systemic change, the study highlighted the parallel need for a top-down strategy that is essential for scaling and achieving a broader impact, while mitigating tensions between governance and leadership.

While the community achieved many notable successes, it also operated within a set of inherent tensions, including professional controversy and competition between healthcare organizations. One of the most significant tensions was the complicated dynamic created by the MoH dual role as both convener for innovation and health system regulator. As the primary initiator and formal leader of the community, the MoH brought legitimacy, resources, and the ability to connect grassroots initiatives with national decision-making channels. However, its leadership also meant that the community was not entirely self-directed. The MoH held an explicit agenda to steer digital transformation in line with its broader policy objectives, which could influence which projects were prioritized, how resources were allocated, and the speed at which ideas were scaled or not. This dynamic sometimes created unease among participants, particularly when bottom-up innovation proposals did not align with the MoH's strategic direction, and in some cases led to declining engagement and inactive groups.

Further, our study revealed the importance of developing digital resilience in understanding technology use in the present and future. Our findings emphasis the need to go beyond the use of technologies within specific service or clinical contexts, to building a collective form of resilience. This multi-faceted community-level resilience emphasizes the collaborative dynamics necessary for enhancing the healthcare system's capacity to withstand and adapt to disruptions. It extends beyond a solely individual-focused view of digital resilience, suggesting that the healthcare system's resilience depends not only on individual adaptability but also on the strength and flexibility of its professional networks.

The study demonstrates that digital resilience is not a static attribute, but an emergent capability shaped through social learning, collaborative governance, and digital affordances within an online environment. These processes enabled the community to function as a strategic platform for experimentation, shared problem-solving, and policy engagement, thereby strengthening the healthcare system's ability to respond flexibly to future disruptions.

## 6 Conclusions

This study explored how a digitally mediated professional community functioned as a platform for developing system-wide digital resilience. By examining the online community's position in reshaping professional roles, enhancing cross-organizational collaboration, and fostering a culture of innovation, we demonstrate that digital resilience is not a static trait but an emergent, co-constructed capability that spans individuals, institutions, and digital infrastructures.

Our findings highlight three interrelated mechanisms through which the online community supported systemic transformation: (1) enabling professionals to operate beyond traditional disciplinary boundaries, facilitating digitally integrated care and workforce agility; (2) creating neutral, virtual spaces for cross-organizational collaboration, even among competitors and regulators; and (3) enhancing an innovation culture, through system level incubators which allowed healthcare actors to co-design new care models leveraging digital tools.

This study also emphasizes the critical interplay between social and digital infrastructures in cultivating long-term system resilience. While much attention in digital health has been directed toward tools and platforms, our findings demonstrate that social factors, such as relationships, trust, norms, and collaborative practices sustained through the online community, is also vital for digital resilience. Importantly, this type of digitally enabled collaboration does not rely solely on formal mandates or predefined hierarchies but thrives through voluntary participation, shared values, and mutual accountability. As such, the study underscores the need to conceptualize resilience not only as a technological capacity but as a deeply sociotechnical achievement.

For policymakers and health system leaders, this study offers a model for nurturing digital resilience through investment in participatory structures that blend digital platforms with collaborative practices. Such infrastructures can ensure that healthcare systems remain adaptive, inclusive, and future-ready amidst growing uncertainty and complexity. However, it is important to note that the Israeli healthcare context, characterized by four dominant HMOs operating under a universal national insurance law and a central Ministry of Health with regulatory and financing authority, which provides a relatively coordinated environment for national-level initiatives, is a specific and unique case. In more fragmented, privatized, or decentralized healthcare systems, establishing similar cross-organizational communities may require different governance structures, incentive mechanisms, or technological infrastructures. Nonetheless, we believe that the mechanisms identified in the study, including multidisciplinary collaboration, cross-organization partnerships, and innovation scaling, are likely transferable to other contexts, though their implementation would need to be adapted to take account of specific local structural and policy environments.

Future research could extend this work by examining how similar communities function in different national and industry contexts or how digital resilience unfolds over longer time horizons, facing multiple crises. Nonetheless, this study provides a foundational understanding of how online professional communities can act as strategic assets for digital resilience and health transformation at scale.

## Data Availability

The raw data supporting the conclusions of this article will be made available by the corresponding author upon reasonable request.
